# Metabolic Effect of an Oriental Herbal Medicine on Obesity and Its Comorbidities with Transcriptional Responses in Diet-Induced Obese Mice

**DOI:** 10.3390/ijms18040747

**Published:** 2017-04-01

**Authors:** Ji-Young Choi, Ye Jin Kim, Su-Jung Cho, Eun-Young Kwon, Ri Ryu, Myung-Sook Choi

**Affiliations:** 1 Department of Food Sciences and Nutrition, Kyungpook National University, 1370 Sankyuk Dong Puk-Ku, Daegu 702-701, Korea; jyjy31@hanmail.net (J.-Y.C.); freewilly59@hanmail.net (Y.J.K.); chocrystalhihi@hanmail.net (S.-J.C.); savage20@naver.com (E.-Y.K.); sangsang0119@gmail.com (R.R.); 2 Center for Food and Nutritional Genomics Research, Kyungpook National University, 1370 Sankyuk Dong Puk-Ku, Daegu 702-701, Korea

**Keywords:** herbal medicine, obesity, oxidative phosphorylation, RNA-seq, transcriptome

## Abstract

Taeeumjowuitang (TJ) is an alternative herbal medicine that has been used to treat obesity in Korea. The molecular mechanisms involved in TJ-induced anti-obesity effects have not yet been determined. The aim of the current study was to elucidate the effects of TJ on obesity and metabolic syndrome, by analyzing the transcriptional and metabolic responses to TJ treatment. C57BL/6J mice were fed a high-fat or high-fat + 3% (*w*/*w*) TJ diet for 12 weeks. Their phenotypic characteristics were measured and the anti-obesity mechanism was elucidated, based on the RNA sequencing (RNA-seq) transcriptomic profiles in an animal model of obesity. TJ treatment ameliorated insulin resistance, dyslipidemia, and hepatic steatosis in high-fat diet-induced obese mice, with a simultaneous reduction in body weight gain by enhancing energy expenditure and suppressing adiposity. An analysis of the global transcriptional changes by RNA-seq revealed that TJ upregulated mitochondrial oxidative phosphorylation-associated genes in epididymal white adipose tissue (eWAT), suggesting an enhanced mitochondrial function after TJ treatment. Moreover, TJ effectively attenuated the high-fat diet-induced inflammatory response through transcriptional changes in eWAT. Our findings provide some mechanistic insights into the effects of TJ, an alternative oriental medicine, in the treatment of obesity and its comorbidities. They demonstrate that metabolic and transcriptional responses to diet-induced obesity with TJ treatment were desirable in adipose tissue metabolism.

## 1. Introduction

Obesity is a metabolic disorder characterized by an excessive accumulation of body fat due to the imbalance between energy intake and expenditure. It is a major factor contributing to the development of pathological conditions such as dyslipidemia, cardiovascular disease, type 2 diabetes mellitus, fatty liver, and even some cancers [1,2]. Adipose tissue plays a major role in energy homeostasis and is not only a storage site for excessive energy, but also an endocrine organ, secreting hormones, cytokines, and proteins that affect whole-body homeostasis [3]. White adipose tissue (WAT) is involved in obesity and its complications. Specifically, chronic inflammation in the WAT plays a pivotal role in the development of obesity-related metabolic diseases [4]. In addition, enlarged WAT leads to the dysregulated secretion of adipokines and promotes the release of free fatty acids (FFAs) into the circulatory system from adipocytes; the released FFAs and pro-inflammatory adipokines also play a key role in the development of obesity-related metabolic disturbances, especially insulin resistance [5,6]. Thus, unveiling the molecular function of adipose tissue is critical for a better overall understanding of energy metabolism and inflammation response.

In many insulin-resistant states, including obesity and type 2 diabetes, the electron transport chain function is dysregulated [7]. Mitochondrial mass and its activity decreased in the WAT of obese mice [8], and the expression of oxidative phosphorylation (OXPHOS)-related genes was markedly downregulated in the WAT of diet-induced obese and *db*/*db* mice [9]. In the WAT of obese humans, the expression and activity of the components of OXPHOS were downregulated; this expression pattern was correlated with the degree of obesity [10]. In addition, the decreased mitochondrial capacity in adipocytes has been suggested to alter insulin sensitivity and function [11]. Thus, the contribution of adipocyte mitochondria to whole-body energy metabolism may depend on the mitochondrial OXPHOS capacity in the adipose tissue [12].

Taeeumjowuitang (TJ), composed of water extracts from eight plants, is a traditional Korean herbal medicine. The eight plant extracts in TJ are: *Coicis Semen*, *Castaneae Semen*, *Raphani Semen*, *Schizandrae Fructus*, *Platycodi Radix*, *Radix Ophiopogonis*, *Acori Graminei Rhizoma*, and *Ephedrae Herba*. The anti-obesity effect of TJ has been demonstrated in some animal and human studies [13,14,15,16]. However, the mechanism attributable to this anti-obesity effect has not yet been elucidated. In the present study, we investigated the possible mechanisms of the anti-obesity effect of TJ by focusing on its phenotypic and transcriptional responses in an obesogenic animal model. This is the first report to integrate the efficacy of TJ with a fat tissue transcriptome obtained from RNA sequencing (RNA-seq). The metabolic effects of TJ were evaluated using interactive pathway analysis (IPA) to identify the adipocyte-specific molecular effects. 

## 2. Results

### 2.1. Taeeumjowuitang (TJ) Lowered Both Body and White Adipose Tissue (WAT) Mass, and Improved Fat Tissue Morphology in Diet-Induced Obese (DIO) Mice

**Figure 1:**
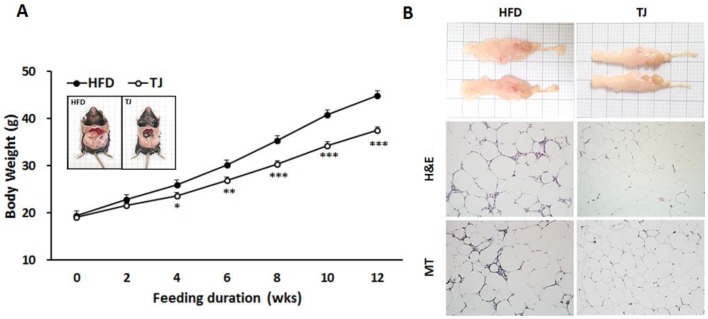
(**A**) Changes in body weight over 12 weeks; (**B**) Hematoxylin and eosin staining (H & E; upper panel) and Masson’s trichrome staining (MT; lower panel) of epididymal adipocytes (magnification 200×) from high-fat diet (HFD)-fed, and Taeeumjowuitang (TJ)-supplemented C57BL/6J mice. The data are shown as mean ± SEM (*n* = 10). TJ vs. HFD; * *p* < 0.05, ** *p* < 0.01, *** *p* < 0.001. HFD, 60% kcal from fat; TJ, HFD + Taeeumjowuitang (3%, *w*/*w*).

After 12 weeks, body weight was significantly lower in the TJ group than in the high-fat diet (HFD) group (Figure 1A). Energy intake was not significantly different between groups; therefore, the food efficiency ratio (FER) was significantly lower in the TJ group than in the HFD group (Table 1). Similar to the trends observed in body weight, liver weight per 100 g body weight was significantly lower in the TJ-treated group than in the HFD group. The significant reductions in kidney and muscle weights observed in the HFD group were reversed upon treatment with TJ (Table 1). When WAT weight was expressed as g/100 g body weight, perirenal, mesenteric, and interscapular WAT deposits were significantly lower in the TJ group than in the HFD group, by 29.1%, 49.6%, and 21.9%, respectively, while the weights of visceral and total WAT deposits decreased by 18.5% and 18%, respectively (Table 1). In addition, the epididymal adipocyte size in the TJ mice was visibly smaller than in the HFD-fed mice. Masson’s trichrome staining of epididymal WAT (eWAT) revealed visible morphological evidence of fibrosis in the HFD group, while no signs of fibrotic changes were identified in the TJ group (Figure 1B).

### 2.2. TJ Improved Lipid Profiles in Plasma and Liver, and Hepatic Tissue Morphology, While Altering Hepatic Lipid Regulating Enzyme Activities in DIO Mice

**Figure 2:**
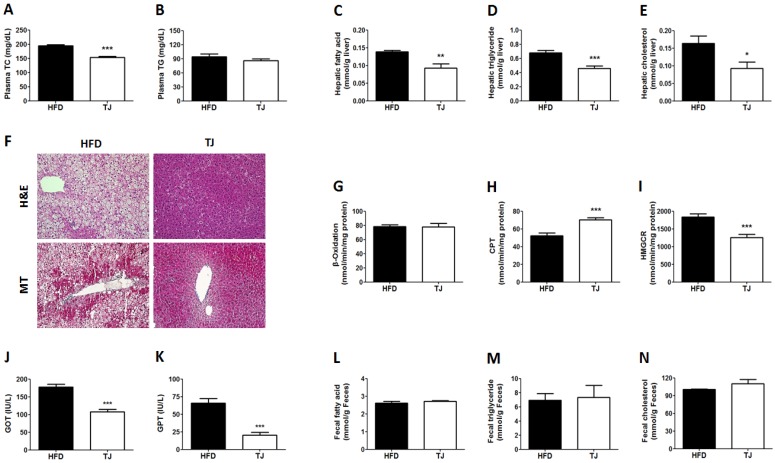
(**A**) Plasma total-cholesterol concentration; (**B**) Plasma triglyceride concentration; (**C**–**E**) Hepatic lipid contents; (**F**) Hematoxylin and eosin staining (H & E; upper panel), and Masson’s trichrome staining (MT; lower panel) of livers (magnification 200×); (**G–I**) Hepatic lipid regulating enzyme activities; (**J**) Plasma glutamic oxaloacetic transaminase; (**K**) Plasma glutamic pyruvic transaminase; and (**L**–**N**) Fecal lipid content in high-fat diet (HFD)-fed, and Taeeumjowuitang (TJ)-supplemented C57BL/6J mice. The data are shown as mean ± SEM (*n* = 10). TJ vs. HFD; * *p* < 0.05, ** *p* < 0.01, *** *p* < 0.001. HFD, 60% kcal from fat; TJ, HFD + Taeeumjowuitang (3%, *w*/*w*).

The plasma total-cholesterol level was significantly lower in the TJ group than in the HFD group at the end of the experimental period (Figure 2A). There was no significant difference in the plasma triglyceride level between groups at the end of the experimental period (Figure 2B). The hepatic fatty acid, triglyceride, and cholesterol contents in the TJ group were significantly lower when compared to those in the HFD group (Figure 2C–E). Hepatic tissue morphology also showed a decreased accumulation of hepatic lipid droplets in the TJ groups (Figure 2F). Masson’s trichrome (MT) staining of the liver revealed no fibrotic change in the TJ group, whereas fibrosis, indicated by blue staining, was observed around the vessels in the HFD group (Figure 2F). Of the two enzymes involved in fatty acid oxidation, the activity of β-oxidation was not significantly different between the HFD and TJ groups, whereas the activity of carnitine palmitoyl-CoA transferase (CPT) increased in the TJ treatment group (Figure 2G,H). The activity of the hepatic cholesterol-regulating enzyme, HMG-CoA reductase (HMGCR), markedly decreased in the TJ group relative to that in the HFD group (Figure 2I). Furthermore, the levels of plasma GOT and GPT (two hepatic toxicity markers) were significantly decreased by TJ treatment (Figure 2J,K).

### 2.3. TJ Reduced Insulin Resistance and Plasma Glucose in DIO Mice

**Figure 3:**
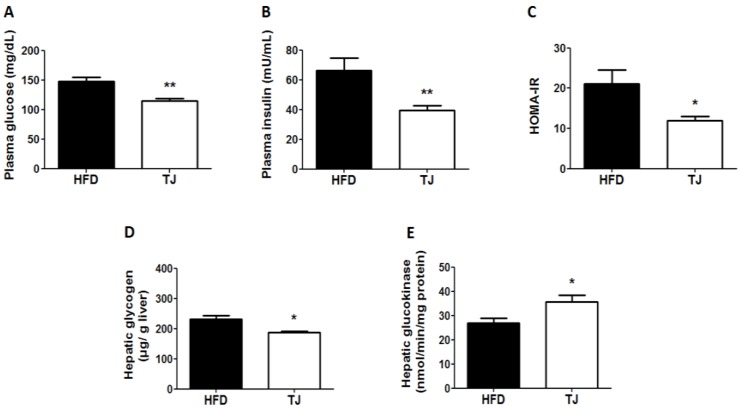
Changes in the (**A**) Fasting plasma glucose concentration; (**B**) Plasma insulin concentration; (**C**) Insulin resistance, according to the homeostasis model assessment for insulin resistance (HOMA-IR); (**D**) Hepatic glycogen content; and (**E**) Hepatic glucokinase enzyme activity in high-fat diet (HFD)-fed, and Taeeumjowuitang (TJ)-supplemented C57BL/6J mice. The data are shown as mean ± SEM (*n* = 10). TJ vs. HFD; * *p* < 0.05, ** *p* < 0.01. HFD, 60% kcal from fat; TJ, HFD + Taeeumjowuitang (3%, *w*/*w*).

The plasma glucose and insulin concentrations significantly decreased with TJ treatment after 12 weeks, compared to those in the HFD group (Figure 3A,B). HOMA-IR was significantly lower in the TJ group (Figure 3C), indicating decreased insulin resistance. The HFD-induced increase in hepatic glycogen was attenuated by TJ treatment (Figure 3D), while glucokinase activity significantly increased in the TJ group (Figure 3E).

### 2.4. TJ Attenuated the Level of Plasma Adipokines and Inflammatory Cytokines in DIO Mice

The plasma leptin and resistin levels were remarkably lower in TJ-treated mice compared to those in the HFD group (Table 2). In contrast, the plasma adiponectin level was significantly elevated in the TJ group when compared to the HFD group. Furthermore, the plasma levels of plasminogen activator inhibitor 1(PAI-1), interferon gamma (IFN-γ), and monocyte chemotactic protein 1 (MCP-1) were significantly decreased in the TJ group compared to those in the HFD group (Table 2).

### 2.5. TJ Enhances Energy Expenditure in DIO Mice

**Figure 4:**
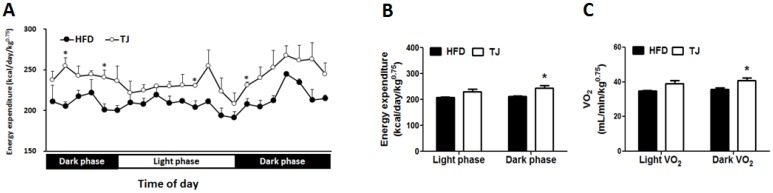
(**A**,**B**) Energy expenditure; and (**C**) Oxygen consumption (VO_2_) in high-fat diet (HFD)-fed and Taeeumjowuitang (TJ)-supplemented C57BL/6J mice. The data are shown as mean ± SEM (*n* = 10). TJ vs. HFD; * *p* < 0.05. HFD, 60% kcal from fat; TJ, HFD + Taeeumjowuitang (3%, *w*/*w*).

TJ treatment resulted in a significant augmentation of the energy expenditure, relative to that in the HFD group, during the dark phase (Figure 4A,B). Furthermore, TJ-treated diet-induced obese (DIO) mice displayed a higher oxygen consumption (VO_2_) during the dark phase (Figure 4C).

### 2.6. TJ Induces the Protein Expression Related to AMPK Pathway and Mitochondrial Function in eWAT

**Figure 5:**
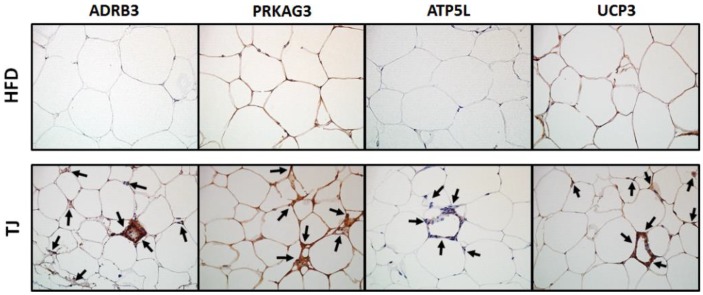
Immunohistochemical staining of epididymal adipocytes (magnification 400×) from high-fat diet (HFD)-fed, and Taeeumjowuitang (TJ)-supplemented C57BL/6J mice. HFD, 60% kcal from fat; TJ, HFD + Taeeumjowuitang (3%, *w*/*w*); ADRB3, beta-3 adrenergic receptor; PRKAG3, protein kinase, AMP-activated, gamma 3 non-catatlytic subunit; ATP5L, ATP synthase, H^+^ transporting, mitochondrial Fo complex subunit G; UCP3, uncoupling protein 3.

Immunohistochemistry analysis showed that the eWAT depot from the TJ group contained more beta-3 adrenergic receptor (ADRB3)-positive cells than that from the HFD group (Figure 5). Furthermore, eWAT in TJ-treated mice revealed a substantial increase in the abundance of multilocular-, PRKAG3- (AMPK gamma 3), ATP5L-, and uncoupling protein 3 (UCP3)-expressing adipocytes when compared with HFD-fed mice (Figure 5).

### 2.7. TJ Modulates Transcriptomic Networks of eWAT in DIO Mice

**Figure 6:**
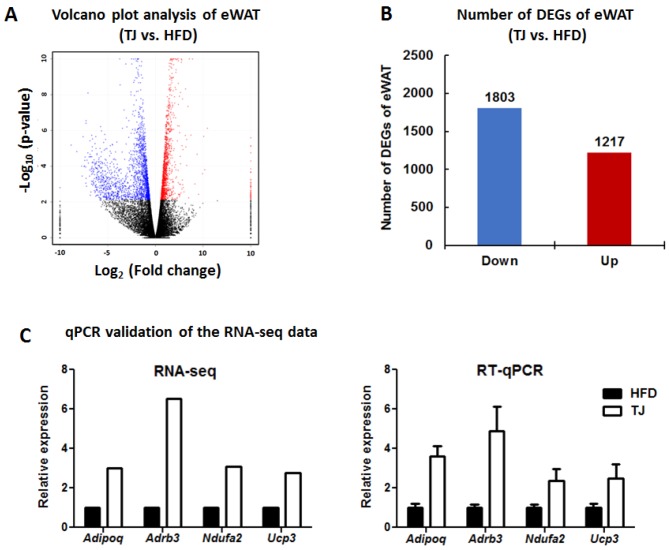
(**A**) Volcano plot analysis of the epididymal white adipose tissue (eWAT) (TJ vs. HFD); (**B**) The number of differentially expressed genes (DEGs) in the eWAT (TJ vs. HFD); and (**C**) Validation of the selected DEGs by quantitative RT-PCR in high-fat diet (HFD)-fed and Taeeumjowuitang (TJ)-supplemented C57BL/6J mice. The data are shown as mean ± SEM. HFD, 60% kcal from fat; TJ, HFD + Taeeumjowuitang (3%, *w*/*w*).

**Figure 7:**
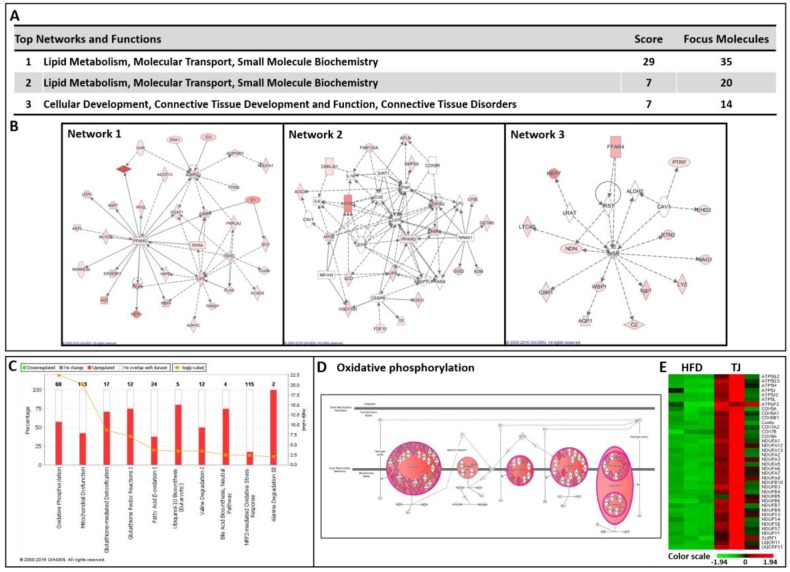
(**A**,**B**) Top three significant networks; (**C**) Significant canonical pathways; (**D**) Transcriptional patterns related to the oxidative phosphorylation pathway (**E**) A heat map of the genes related to oxidative phosphorylation in the epididymal white adipose tissue (eWAT) of Taeeumjowuitang (TJ)-treated mice versus high-fat diet (HFD)-fed mice. The significant networks and pathways were identified by upregulated differentially expressed genes (DEGs) in the eWAT of TJ versus HFD, using Ingenuity Pathway Analysis (IPA). A red-green color scale depicts normalized expression levels (based on Z-scores) in FPKM values. The lines between genes represent known interactions (solid lines represent direct interactions; dashed lines represent indirect interactions). White objects denote measured genes with no significant change, red shading denotes upregulation, and green shading downregulation in expression relative to the HFD. The darker the color, either red or green, the higher the fold change.

When the DEGs in the eWAT were examined, 3020 DEGs (1217 up- and 1803 down-regulated genes) were differentially expressed in the TJ group compared to the number seen in the HFD group (*q*-value < 0.05 and fold change ≥ 1.5) (Figure 6A,B). In order to validate RNA-seq data, we performed RT-qPCR. We selected genes which are involved in the AMPK pathway (*Adipoq* and *Adrb3*) and mitochondrial function (*Ndufa2* and *Ucp3*) in the DEGs list (TJ vs. HFD). As a result, the RNA-seq- and RT-qPCR-determined expression levels were in good agreement (Figure 6C). The 3020 DEGs in the TJ vs. HFD group were used for Ingenuity Pathway Analysis (IPA), to identify significant molecular networks and canonical pathways underlying the gene expression profiles altered by TJ. For the 1217 up-regulated DEGs mapped to IPA (genes not mapped to the IPA database were excluded in our pathway analysis), the top-ranked networks (score ≥ 5) and canonical pathways (*p*-value < 0.05) in eWAT are shown in Figure 7. Of the top three networks, “Lipid Metabolism, Molecular Transport, Small Molecule Biochemistry” was the highest rated network, with 35 focus molecules and a significance score of 29 (Figure 7A,B). This indicates that TJ may regulate lipid metabolic processes in the eWAT. Of 38 canonical pathways (*p*-value < 0.05), the top 10 pathways in the TJ vs. HFD groups are shown in Figure 7C. Based on IPA, the most significant pathway identified among canonical pathways was “Oxidative Phosphorylation (OXPHOS)” (Figure 7C), and the majority of OXPHOS-associated gene components were upregulated in eWAT upon TJ treatment (Figure 7D). In addition, glutathione-mediated detoxification, fatty acid β-oxidation, and degradation pathways of amino acids, including valine, alanine, leucine, tryptophan, tyrosine, and isoleucine, were identified as significant canonical pathways (Figure 7C and Supplementary Table S3).

**Figure 8:**
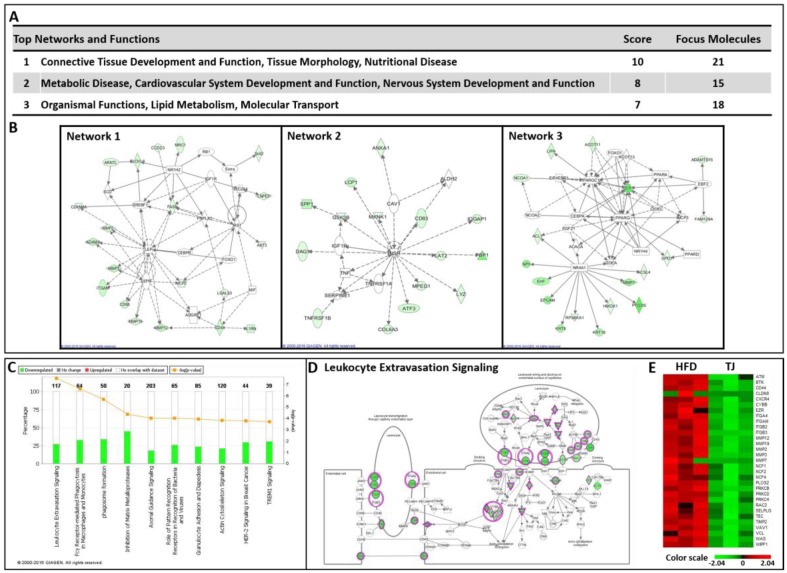
(**A**,**B**) Top three significant networks; (**C**) Significant canonical pathways; (**D**) Transcriptional patterns related to the leukocyte extravasation signaling; (**E**) A heat map of the genes related to leukocyte extravasation signaling in the epididymal white adipose tissue (eWAT) of Taeeumjowuitang (TJ)-treated mice versus high-fat diet (HFD)-fed mice. The significant networks and pathways were identified by downregulated differentially expressed genes (DEGs) in the eWAT of TJ versus HFD, using Ingenuity Pathway Analysis (IPA). A red-green color scale depicts normalized expression levels (based on Z-scores) in FPKM values. The lines between genes represent known interactions (solid lines represent direct interactions; dashed lines represent indirect interactions). White objects denote measured genes with no significant change, red shading denotes upregulation, and green shading downregulation in expression relative to the HFD. The darker the color, either red or green, the higher the fold change.

Meanwhile, the top three networks identified by down-regulated DEGs were associated with the networks “Connective Tissue Development and Function, Tissue Morphology, Nutritional Disease”, “Metabolic Disease, Cardiovascular System Development and Function, Nervous System Development and Function”, and “Organismal Functions, Lipid Metabolism, Molecular Transport” (Figure 8A,B). Of these three networks, “Connective Tissue Development and Function, Tissue Morphology, Nutritional Disease” was the highest rated network, with 21 focus molecules and a significance score of 10 (Figure 8A). We also identified canonical pathways associated with immune responses, including leukocyte extravasation, phagocytosis in macrophages and monocytes, phagosome formation, and many genes involved in inflammatory signaling in the eWAT of TJ vs. HFD (Figure 8C–E and Supplementary Table S4).

## 3. Discussion

In the current study, we evaluated the effect of TJ and the potential mechanisms underlying metabolic regulation by using RNA-seq transcriptomic profiles in a diet-induced obesity model. As expected, TJ treatment attenuated HFD-induced obesity, dyslipidemia, hepatic steatosis, insulin resistance, and inflammatory response. The present study also showed the multiple effects of TJ involved in ameliorating metabolic disturbances in obese mice.

In general, obesity is a key risk factor in the development of metabolic diseases [17]. Average daily weight gains for the 12-week experimental period were significantly lower in the TJ group than in the HFD group, despite unaltered energy intake between TJ and HFD groups. Given these results, the FER was significantly lower in TJ-treated mice. One possible explanation for the lower FER could be the increased energy expenditure during the dark phase upon treatment with TJ, compared to that in the HFD group. This was also supported by the measurement of a higher oxygen consumption (VO_2_) in the TJ group during the dark phase. TJ treatment reversed the increased body fat mass and adipocyte hypertrophy observed in HFD mice. In eWAT adipocytes, TJ-treated mice showed no signs of collagen fibril infiltration, which is common in obese mice. 

Another possible explanation for the observed body fat reduction could be the increased transcriptional response of OXPHOS upon treatment with TJ when compared to the HFD group. According to previous studies, a sustained activation of ADRB3 is displayed in the pronounced remodeling of WAT, which contains mitochondrial biogenesis and the augmentation of the metabolic rate [18]. In this study, the chronic activation of ADRB3 by TJ significantly altered the expression of OXPHOS-related genes, including the electron transport chain, in eWAT. In particular, our data, obtained using IPA, showed that OXPHOS is the most significant canonical pathway among the 38 canonical pathways. A majority of the OXPHOS-associated genes, including NADH dehydrogenase (complex I), ubiquinol-cytochrome c reductase (complex III), cytochrome c oxidase (complex IV), and ATP synthase, were augmented by TJ treatment. Consistent with the present study, most insulin-resistant states are characterized by the dysregulation of electron transport chain function [7]. The expression and activity of the OXPHOS components were downregulated in the WAT of human subjects, correlating with the degree of obesity [10], and the mitochondrial OXPHOS capacity of the adipose tissue was important to whole-body energy metabolism [12]. Therefore, our results suggest that the TJ-induced increase in OXPHOS-related gene expression enhanced the metabolic rate and regulated energy metabolism in eWAT. In *db*/*db* and diet-induced obese mice, the expression of OXPHOS-related genes was markedly decreased, compared to that in normal mice [9].

It is likely that increased mitochondrial OXPHOS-associated gene expression in the eWAT of TJ-treated mice contributed to the improved glucose metabolism in this study. Adiponectin has been shown to act as an insulin sensitizer [19,20]. Plasma adiponectin, a controller of energy homeostasis, was significantly elevated by TJ treatment, with a concomitant increase in the mRNA expression of *Adipoq* (adiponectin) in eWAT. Moreover, the activity of hepatic glucokinase of the TJ group was significantly higher than that in the HFD group, with a decrease in the hepatic glycogen content. It is plausible that TJ improves insulin resistance through increases in hepatic glucokinase activity and the upregulation of *Adipoq* and OXPHOS-related gene expression in eWAT.

Consistent with reduced fat mass, TJ-treated mice exhibited significant improvements in adipokine secretion, including leptin, resistin, cytokines, and chemokines, compared with the HFD mice. Leptin, the satiety hormone, regulates food intake and energy expenditure. Resistin promotes both inflammation and insulin resistance in animal models [21]. These two adipokines significantly decreased with TJ treatment. In the obese state, the adipocyte is integral to the development of obesity-induced inflammation, by increasing the secretion of various pro-inflammatory chemokines and cytokines [22]. The inflammatory markers such as PAI-1, IFN-γ, and MCP-1 in the TJ group significantly decreased the HFD group level. In addition, according to IPA, TJ supplementation attenuated the diverse signaling pathways associated with immune responses, including the pathways related to leukocyte extravasation, phagocytosis in macrophages and monocytes, communication between cells of innate and adaptive immunity, and many types of inflammatory signaling in the eWAT of DIO mice.

The chronic administration of an HFD can cause nonalcoholic steatohepatitis (NASH) in animal models and long-standing NASH may proceed to liver cirrhosis [23]. A histological examination of liver tissue from TJ-treated DIO mice revealed a reduction in lipid droplets compared with HFD control mice, indicating the amelioration of hepatic steatosis. Consistent with this histology, marked decreases in hepatic FA, triglyceride, and cholesterol contents, and increased hepatic activities of CPT, were seen after TJ treatment, with simultaneous decreases in HMGCR activity, which is the primary means for controlling cholesterol biosynthesis. Part of this result was consistent with a preceding study that demonstrated that hepatic triglyceride content was significantly lowered by TJ [24]. Moreover, decreased plasma GOT and GPT levels were measured in TJ-treated mice, indicative of the reduced liver damage induced by HFD.

In summary, the data obtained from the present study indicates that TJ treatment can improve or suppress diet-induced obesity and modulate obesity-associated metabolic disorders, such as insulin resistance, dyslipidemia, and fatty liver disease. This modulation occurs partly through an increase in energy expenditure and regulation of lipid, glucose, and inflammatory responses. Overall, metabolic and transcriptional responses to diet-induced obesity with TJ treatment were desirable. In particular, TJ increased the expression of mitochondrial OXPHOS-associated genes in eWAT, suggesting enhanced mitochondrial function after TJ treatment. Moreover, TJ was an effective regulator in the attenuation of the high-fat diet-induced inflammatory response through transcriptional changes in eWAT. Taken together, the present findings provide important mechanistic insights into the anti-obesity effects and amelioration of metabolic complications provided by treatment with the alternative oriental medicine known as TJ.

## 4. Materials and Methods

### 4.1. Animals

Thirty male C57BL/6J mice (four-week-old) were obtained from The Jackson Laboratory (Bar Harbor, ME, USA). All mice were individually housed under a constant temperature (24 °C) and 12-h light/dark cycle. The mice were fed a normal chow diet for one week after arrival, as an acclimatization period. At five weeks of age, they were randomly divided into two groups of 10 mice per group and fed either a high-fat diet (HFD) or HFD + 3% (*w*/*w*) TJ for 12 weeks (Table S2). The HFD (TD06414, Harlan, Madison, WI, USA) contained 60.3 kcal% fat, 18.4 kcal% protein, and 21.3 kcal% carbohydrate. TJ extracts were obtained from I-world Pharm (Incheon, Korea). The dose of TJ was determined based on the Ministry of Food and Drug Safety (MFDS, formerly known as the Korea Food & Drug Administration or KFDA, Cheongju-si, Korea) guidelines. The human TJ dose, 9 g/day for adults, was converted to a mouse dose using the body surface area normalization method [25]. The mice were provided free access to food and distilled water, while body weight and blood glucose levels were measured every 1 to 2 weeks. At the end of the diet period, all mice were anesthetized with isoflurane after a 12-h fast. Blood was taken from the inferior vena cava for the determination of glucose, plasma lipid, and hormone concentrations. The liver and adipose tissue were removed, rinsed with physiological saline, weighed, immediately frozen in liquid nitrogen, and stored at −70 °C until analysis. The animal study protocols were approved by the Ethics Committee of Kyungpook National University, Daegu, Korea (KNU 2012-136, 04 December 2012).

### 4.2. Measurements of Energy Expenditures

Energy expenditure was measured using an indirect calorimeter (Oxylet; Panlab, Cornella, Spain). The mice were placed into individual metabolic chambers at 25 °C, with free access to food and water. O_2_ and CO_2_ analyzers were calibrated with high-purity gas. Oxygen consumption and carbon dioxide production were recorded at 3 min intervals, using a computer-assisted data acquisition program (Chart 5.2; AD Instrument, Sydney, Australia) over a 24-h period, and the data were averaged for each mouse. Energy expenditure (EE) was calculated according to the following formula:

EE (kcal/day/body weight^0.75^) = VO_2_ × 1.44 × [3.815 + (1.232 × VO_2_/VCO_2_)]

### 4.3. Analysis of Plasma, Hepatic, and Fecal Lipids

Enzymatic assays to determine the plasma total-cholesterol and triglyceride levels were performed using kits purchased from Asan Pharm Co. (Seoul, Korea). Hepatic and fecal lipids were extracted according to the methods of Folch [26]. Then, both the cholesterol and triglyceride levels were determined using the same enzymatic kit used for the plasma analyses. The hepatic and fecal fatty acid (FA) levels were measured using the Wako enzymatic kit (Wako Chemicals, Richmond, VA, USA).

### 4.4. Plasma Glutamic Oxaloacetic Transaminase (GOT) and Glutamic Pyruvic Transaminase (GPT) Activities

GOT and GPT activities were measured using commercially available kits (Asan Pharm Co., Seoul, Korea).

### 4.5. Plasma Glucose and Insulin Resistance Index

The plasma glucose level was measured using a commercially available kit (Asan Pharm Co., Seoul, Korea). The homeostasis model assessment for insulin resistance (HOMA-IR) was calculated using the following formula:

HOMA-IR = [fasting insulin concentration (mU/L)] × [fasting glucose concentration (mg/dL) × 0.05551]/22.5

### 4.6. Plasma Hormones, Adipokines, and Proinflammatory Cytokines

Plasma concentrations of insulin and adipokines (leptin, resistin, and plasminogen activator inhibitor 1 (PAI-1)) were quantified using a multiplex detection kit (171-F7001M, Bio-Rad, Hercules, CA, USA) according to the manufacturer’s protocol. Plasma concentrations of adiponectin and plasma cytokines (interferon γ (IFN-γ), and monocyte chemoattractant protein 1 (MCP-1)) were quantified using a detection kit (171-F7002M, Bio-Rad, Hercules, CA, USA) and multiplex detection kit (M60-009RDPD, Bio-Rad), respectively, according to the manufacturer’s instructions.

### 4.7. Hepatic enzyme Activities and Glycogen Concentration

Fatty acid β-oxidation and carnitine palmitoyltransferase (CPT) activities were measured according to previously described protocols [27,28]. Microsomal HMG-CoA reductase (HMGCR) activity was measured using [^14^C]-HMG-CoA and [^14^C]-Oleoyl CoA as substrates [29]. The hepatic glycogen concentration was determined as previously described [30].

### 4.8. Histological Analysis of Epididymal WAT (eWAT) and the Liver

The liver and eWAT were excised from each mouse, fixed in 10% (*v*/*v*) paraformaldehyde in phosphate buffered saline (PBS), and embedded in paraffin for staining with hematoxylin and eosin (H & E) and Masson’s trichrome dye. The stained slices were examined under an optical microscope (Zeiss Axioscope) at 200× magnification [31]. For immunohistochemistry, paraffin-embedded sections were incubated at 4 °C with anti-ADRB3, anti-ATP5L, anti-PRKAG3, or anti-UCP3 (Abcam, Cambridge, MA, USA), followed by detection using the ABC Vectastain-Elite kit (Vectastain ABC kit, Vector Labs, Burlingame, CA, USA) according to the manufacturer’s instructions.

### 4.9. RNA Preparation, Library Preparation, and RNA-Seq

The eWAT was collected from three mice randomly selected from each of the HFD and TJ groups. The total RNA was extracted from eWAT using TRIzol Reagent (Invitrogen Life Technologies, Carlsbad, CA, USA) according to the manufacturer’s instructions. After synthesizing cDNA libraries, the qualities of the cDNA libraries were evaluated using an Agilent 2100 BioAnalyzer (Agilent, Santa Clara, CA, USA). The cDNA libraries were quantified using the KAPA Library Quantification Kit (Kapa Biosystems, Wilmington, MA, USA). After cluster amplification of the denatured templates, samples in flow cells were sequenced as paired-end polymers (2 × 100 bp) using Illumina HiSeq2500 (Illumina, San Diego, CA, USA)

### 4.10. Preprocessing of the RNA-Seq Data

Low-quality reads were filtered out according to the following criteria: reads containing >10% of skipped bases (marked as N’s), reads containing >40% of bases whose quality scores were <20, and reads whose average quality score was <20. The filtering process was performed using in-house scripts. The remaining reads were mapped onto the human reference genome (Ensembl release 72), using the aligner software STAR v.2.3.0e [32]. The gene expression levels were measured by Cufflinks v2.1.1 [33], using the gene annotation database of Ensembl release 72. The noncoding gene regions were removed by means of the mask option. To improve the accuracy of measurement, “multiread correction” and “frag bias-correct” options were used. All other options were set to default values.

### 4.11. RT-qPCR

The total RNA (1 μg) was reverse-transcribed into cDNA using the QuantiTect^®^ reverse transcription kit (Qiagen, Hilden, Germany). The mRNA expression was then quantified by real-time quantitative PCR (RT-qPCR), using the SYBR green PCR kit (Qiagen, Hilden, Germany) and the CFX96TM real-time system (Bio-Rad, Hercules, CA, USA). Gene-specific mouse primers were used as presented in Supplementary Table S1. Cycle threshold (Ct) data were normalized using glyceraldehyde-3-phosphate dehydrogenase (GAPDH), and the relative gene expression was calculated using the 2^−∆∆*C*t^ method [34].

### 4.12. Differential Transcriptome and Functional Analysis

For differential expression analysis, the data on gene level counts were generated using HTSeq-count v0.5.4p3 [35]. Using the resulting read count data, differentially expressed genes (DEGs) were identified using the R software package TCC [36]. The TCC package uses robust normalization strategies to compare tag count data. Normalization factors were calculated using the iterative DEGES/edgeR method. The Q value was calculated from the *p* value using the p.adjust function in the R package and the default settings. DEGs were identified based on a *q* value threshold less than 0.05. K-means clustering was performed in the Bioinformatics Toolbox of MATLAB R2009a. These RNA-seq data were deposited in the Gene Expression Omnibus (GEO) database (GEO accession number: GSE71586).

### 4.13. Molecular Network and Pathway Analysis

The DEG lists were analyzed using Ingenuity Pathway Analysis software (IPA, Ingenuity^®^ systems, Qiagen, Redwood City, CA, USA). IPA allows for the identification of network interactions and pathway interactions between genes, based on an extensive manually curated database of published gene interactions. We uploaded the genes with a *q* value threshold of less than 0.05 and a fold change in expression of more than 1.5, after HFD, with or without TJ supplementation, and the associated expression value from the RNA-seq data, into IPA. 

### 4.14. Statistical Analysis

The values were expressed as the mean ± standard error of the mean (SEM). Significant differences between the HFD and TJ groups were assessed by Student’s *t* tests and Wilcoxon’s *t* tests using the SPSS v18.0 software (SPSS Inc., Chicago, IL, USA).

## Figures and Tables

**Table 1:** Differences in energy intake, food efficiency ratio, organ weight, and adipose tissue weight from high-fat diet (HFD)-fed and Taeeumjowuitang (TJ)-supplemented C57BL/6J mice.

Parameters	HFD	TJ
Energy Intake (kcal/day)	13.63 ± 0.07	13.33 ± 0.20
FER	0.022 ± 0.001	0.016 ± 0.00 ***
Organ Weights (g/100 g Body Weight)		
Liver	3.96 ± 0.19	3.31 ± 0.18 *
Kidney	0.75 ± 0.02	0.83 ± 0.02 *
Muscle	0.72 ± 0.02	0.85 ± 0.04 **
Adipose Tissue Weights (g/100 g Body Weight)		
Epididymal WAT	5.89 ± 0.19	5.33 ± 0.37
Perirenal WAT	0.96 ± 0.05	0.68 ± 0.06 **
Subcutaneous WAT	3.11 ± 0.24	2.73 ± 0.31
Retroperitoneum WAT	1.53 ± 0.05	1.50 ± 0.09
Mesenteric WAT	2.54 ± 0.14	1.28 ± 0.18 ***
Interscapular WAT	3.14 ± 0.08	2.46 ± 0.18 **
Interscapular BAT	0.42 ± 0.01	0.34 ± 0.03 **
Visceral WAT	10.79 ± 0.16	8.79 ± 0.59 **
Total WAT	17.04 ± 0.31	13.98 ± 0.96 *

Data are Mean ± S.E. (*n* = 10). TJ vs. HFD; * *p* < 0.05, ** *p* < 0.01, *** *p* < 0.001. HFD, High-fat diet (60% kcal from fat); TJ, HFD + Taeeumjowuitang (3%, *w*/*w*); WAT, white adipose tissue; BAT, brown adipose tissue.

**Table 2:** Plasma adipokines and inflammatory cytokines in high-fat diet (HFD)-fed and Taeeumjowuitang (TJ)-supplemented C57BL/6J mice.

Parameters	HFD	TJ
Leptin (ng/mL)	66.05 ± 6.03	20.40 ± 3.70 **
Resistin (ng/mL)	320.21 ± 5.74	197.11 ± 25.67 **
Adiponectin (ug/mL)	6.99 ± 0.47	8.76 ± 0.26 **
PAI-1 (ng/mL)	2.46 ± 0.36	1.50 ± 0.10 *
IFN-γ (pg/mL)	8.12 ± 0.72	6.19 ± 0.48 *
MCP-1 (pg/mL)	281.14 ± 42.81	173.53 ± 23.68 *

Data are Mean ± S.E (*n* = 10). TJ vs. HFD; * *p* < 0.05, ** *p* < 0.01. HFD, High-fat diet (60% kcal from fat); TJ, HFD + Taeeumjowuitang (3%, *w*/*w*); MCP-1, monocyte chemotactic protein 1; IFN-γ, interferon gamma; PAI-1, plasminogen activator inhibitor 1.

## Supplementary Materials

Supplementary materials can be found at www.mdpi.com/1422-0067/18/4/747/s1.

## Abbreviations

ADRB3	beta-3 adrenergic receptor
ATP5L	ATP synthase, H+ transporting, mitochondrial Fo complex subunit G
CPT	Carnitine palmitoyltransferase
DEGs	Differentially expressed genes
DIO	Diet-induced obesity
EE	Energy expenditure
eWAT	Epididymal WAT
FER	Food efficiency ratio
GOT	Glutamic oxaloacetic transaminase
GPT	Glutamic pyruvic transaminase
HFD	High-fat diet
HMGCR	HMG-CoA reductase
HOMA-IR	Homeostasis model assessment for insulin resistance
IFN-γ	Interferon γ
IPA	Ingenuity pathway analysis
MCP-1	Monocyte chemoattractant protein 1
OXPHOS	Oxidative phosphorylation
PAI-1	Plasminogen activator inhibitor 1
PBS	Phosphate buffered saline
PRKAG3	Protein kinase, AMP-activated, gamma 3 non-catatlytic subunit
TJ	Taeeumjowuitang
UCP3	Uncoupling protein 3
