# Supplementary material for: Metabolic Effect of an Oriental Herbal Medicine on Obesity and Its Comorbidities with Transcriptional Responses in Diet-Induced Obese Mice
Source: Int J Mol Sci. 2017 Apr 1;18(4):747. doi: 10.3390/ijms18040747 (PMC5412332; doi:10.3390/ijms18040747)
Supplement: Supplementary file 1 [file ijms-18-00747-s001.pdf]

Metabolic Effect of an Oriental Herbal Medicine on Obesity and Its Comorbidities with Transcriptional Responses in Diet-Induced Obese Mice

Ji-Young Choi [1,2], Ye Jin Kim [1,2], Su-Jung Cho [1,2], Eun-Young Kwon [1,2], Ri Ryu [1,2] and Myung-Sook Choi [1,2,*]

[1] Department of Food Sciences and Nutrition, Kyungpook National University, 1370 Sankyuk Dong Puk-Ku, Daegu 702-701, Korea; jyjy31@hanmail.net (J.-Y.C.); freewilly59@hanmail.net (Y.J.K.); chocrystalhihi@hanmail.net (S.-J.C.); savage20@naver.com (E.-Y.K.); sangsang0119@gmail.com (R.R.)

[2] Center for Food and Nutritional Genomics Research, Kyungpook National University, 1370 Sankyuk Dong Puk-Ku, Daegu 702-701, Korea

* Correspondence: mschoi@knu.ac.kr; Tel.: +82-53-950-6232; Fax: +82-53-950-6229

Ⅰ. Supplementary Methods

Table S1. Primer sequences used for RT-qPCR.

**Primer**	**Sequence**
Adipoq	5’-GGT CTT CTT GGT CCT AAG GGT GAG-3’ (forward)<br>5’-GCG GCT TCT CCA GGC TCT C-3’ (reverse)
Adrb3	5’-TCGACATGTTCCTCCACCAA-3’ (forword)<br>5’-GATGGTCCAAGATGGTGCTT-3’ (reverse)
Ndufa2	5’-GCA CAC ATT TCC CCA CAC TG-3’ (forword)<br>5’-CCC AAC CTG CCC ATT CTG AT-3’ (reverse)
Ucp3	5’-GCACTGCAGCCTGTTTTGCTGA-3’ (forword)<br>5’-ATAGTCAGGATGGTACCGAGCA-3’ (reverse)
Gapdh	5′-ACA ATG AAT ACG GCT ACA GCA ACA G-3′ (forward)<br>5′-GGT GGT CCA GGG TTT CTT ACT CC-3′ (reverse)

Adipoq, adiponectin; *Adrb3*, beta-3 adrenergic receptor; *Ndufa2*, NADH dehydrogenase (ubiquinone) 1 alpha subcomplex, 2; *Ucp3*, uncoupling protein 3; *Gapdh*, Glyceraldehyde 3-phosphate dehydrogenase

Table S2. Diet composition for animal experiment.

Ingredient (g)	HFD	TJ
Casein	265	265
Corn Starch	0	0
Sucrose	90	60
Maltodextrin	160	160
Cellulose	65.6	65.6
Soybean Oil	30	30
Lard	310	310
Mineral Mix[1]	48	48
Vitamin Mix[2]	21	21
Calcium phosphate, Dibasic	3.4	3.4
TBHQ, antioxidant	0	0
L-Cystine	4	4
cholin Bitartrate	3	3
Taeeumjowitang[3]		30
Total (g)	1,000	1,000
Total energy (kcal)	5,100	5,000

HFD, high-fat diet (60% kcal fat); TJ, Taeeumjowitang (HFD + 3% TJ)

[1] AIN-93G- Mineral Mixture, [2]AIN-93G- Vitamin Mixture

[3]Oriental herbal medicine that is a powdered form of multi-plant water extracts. The multi-plants are composed of Coicis Semen 11.25g, Castaneae Semen 11.25g, Raphani Semen 7.5g, Schizandrae Fructus 3.75g, Platycodi Radix 3.75g, Radix Ophiopogonis 3.75g, Acori Graminei Rhizoma 3.75g, and Ephedrae Herba 3.75g.

Ⅱ. Supplementary Tables

Table S3. The significant canonical pathways identified by upregulated differentially expressed genes (DEGs) in Taeeumjowuitang (TJ) versus high-fat-diet (HFD) for the epididymal white adipose tissue. Significant canonical pathways were obtained from Ingenuity Pathway Analysis. TJ= mice fed a high-fat-diet supplemented with Taeeumjowitang, HFD=mice fed a high-fat-diet.

Ingenuity Canonical Pathways	p-value	Ratio	Upregulated	Molecules
Oxidative Phosphorylation	3.E-23	5.74E-01	39/68 (57%)	COX7B,ATP5H,COX6A1,Cox6c,NDUFA7,COX8A,NDUFB5,ATP5L,NDUFA1,NDUFB3,NDUFA2,Atp5e,NDUFB10,NDUFA5,NDUFB9,NDUFS6,ATP5J2,NDUFB6,UQCRFS1,ATP5G3,NDUFS4,NDUFA8,ATP5J,NDUFV1,ATP5G2,COX6B1,NDUFB4,NDUFS7,SURF1,UQCR11,NDUFA13,NDUFA6,NDUFB7,COX5A,COX7A2,NDUFA12,NDUFA3,ATPAF2,NDUFS3
Mitochondrial Dysfunction	6.E-21	4.25E-01	48/113 (42%)	HSD17B10,COX7B,ATP5H,COX6A1,Cox6c,NDUFA7,PRDX5,COX8A,NDUFB5,ATP5L,NDUFA1,NDUFA2,NDUFB3,Atp5e,NDUFB10,NDUFA5,MAOB,NDUFB9,PARK7,NDUFS6,ATP5J2,UQCRFS1,NDUFB6,GPX4,ATP5G3,NDUFA8,NDUFS4,ATP5J,NDUFV1,ATP5G2,COX6B1,NDUFB4,NDUFS7,SURF1,UQCR11,NDUFA13,FIS1,NDUFA6,TXN2,CAT,NDUFB7,COX5A,COX7A2,NDUFA12,NDUFA3,ATPAF2,NDUFS3,PINK1
Glutathione-mediated Detoxification	2.E-09	7.06E-01	12/17 (71%)	GSTZ1,Gstt3,GSTA3,GSTT2/GSTT2B,MGST1,MGST2,GSTM5,GSTM4,Gstt1,GSTO1,MGST3,GSTK1
Glutathione Redox Reactions I	9.E-08	7.5E-01	9/12 (75%)	GSTZ1,GSTT2/GSTT2B,MGST1,MGST2,GPX1,GPX4,Gstt1,MGST3,GSTK1
Fatty Acid β-oxidation I	2.E-04	3.75E-01	9/24 (38%)	Acaa1b,HSD17B10,Eci3,ACAA1,SCP2,SLC27A1,IVD,ECI1,HADH
Ubiquinol-10 Biosynthesis (Eukaryotic)	4.E-04	8E-01	4/5 (80%)	BCKDHA,COQ2,MICAL3,BCKDHB
Valine Degradation I	4.E-04	5E-01	6/12 (50%)	HIBCH,BCAT2,HIBADH,BCKDHA,ACADSB,BCKDHB
Bile Acid Biosynthesis, Neutral Pathway	3.E-03	7.5E-01	3/4 (75%)	CYP27A1,SCP2,HSD3B7

NRF2-mediated Oxidative Stress Response	5.E-03	1.74E-01	20/115 (17%)	GSTA3,AKR7A2,MGST1,GSTM5,RRAS,HSPB8,SOD1,DNAJC15,CLPP,GSTO1,SOD3,GSTT2/GSTT2B,MGST2,CAT,GSTM4,PRKCE,FMO1,CBR1,MGST3,GSTK1
Alanine Degradation III	9.E-03	1E00	2/2 (100%)	GPT,GPT2
Taurine Biosynthesis	9.E-03	1E00	2/2 (100%)	CSAD,CDO1
Alanine Biosynthesis II	9.E-03	1E00	2/2 (100%)	GPT,GPT2
Glutaryl-CoA Degradation	1.E-02	4E-01	4/10 (40%)	HSD17B10,L3HYPDH,HADH,GCDH
Superoxide Radicals Degradation	1.E-02	5E-01	3/6 (50%)	CAT,SOD1,SOD3
LPS/IL-1 Mediated Inhibition of RXR Function	2.E-02	1.56E-01	20/128 (16%)	GSTA3,APOE,MGST1,GSTM5,FMO5,HMGCS2,GSTO1,SOD3,GSTT2/GSTT2B,CHST1,MAOB,MGST2,CAT,GSTM4,FABP4,FMO1,SLC27A1,RXRA,MGST3,GSTK1
Noradrenaline and Adrenaline Degradation	2.E-02	2.94E-01	5/17 (29%)	ADH5,HSD17B10,MAOB,ADH1C,DHRS4
VEGF Family Ligand-Receptor Interactions	2.E-02	2.05E-01	9/44 (20%)	VEGFA,AKT2,PLA2G2D,FLT1,RRAS,PRKCE,VEGFB,NOS3,KDR
Serotonin Degradation	2.E-02	2.5E-01	6/24 (25%)	ADH5,HSD17B10,MAOB,ADH1C,Sult1a1,DHRS4
Leukotriene Biosynthesis	2.E-02	4.29E-01	3/7 (43%)	MGST2,LTC4S,GGT5
Leucine Degradation I	2.E-02	4.29E-01	3/7 (43%)	BCAT2,IVD,MCCC2
Tetrahydrobiopterin Biosynthesis I	2.E-02	6.67E-01	2/3 (67%)	PTS,SPR
Tetrahydrobiopterin Biosynthesis II	2.E-02	6.67E-01	2/3 (67%)	PTS,SPR
Tyrosine Degradation I	2.E-02	6.67E-01	2/3 (67%)	GSTZ1,FAH
Molybdenum Cofactor Biosynthesis	2.E-02	6.67E-01	2/3 (67%)	MOCS2,NFS1
Tryptophan Degradation III (Eukaryotic)	3.E-02	3.08E-01	4/13 (31%)	HSD17B10,L3HYPDH,HADH,GCDH
Xenobiotic Metabolism Signaling	3.E-02	1.42E-01	21/148 (14%)	GSTA3,SRA1,MGST1,GSTM5,RRAS,FMO5,GSTO1,SOD3,GSTT2/GSTT2B,CHST1,MAOB,CES1,MGST2,CAT,GSTM4,PRKCE,FMO1,RXRA,SCAND1,MGST3,GSTK1
Retinol Biosynthesis	4.E-02	2.86E-01	4/14 (29%)	RBP7,CES1,LIPE,PNPLA2
Ethanol Degradation II	4.E-02	2.86E-01	4/14 (29%)	ADH5,HSD17B10,ADH1C,DHRS4
Aryl Hydrocarbon Receptor Signaling	4.E-02	1.57E-01	14/89 (16%)	GSTA3,MGST1,GSTM5,RARG,GSTO1,GSTT2/GSTT2B,MGST2,CCND3,NFIA,GSTM4,RXRA,MGST3,HSPB1,GSTK1
LXR/RXR Activation	4.E-02	1.67E-01	11/66 (17%)	APOE,SCD,FDFT1,LYZ,MLXIPL,TF,VTN,RXRA,HADH,AGT,RBP4
Isoleucine Degradation I	4.E-02	3.33E-01	3/9 (33%)	HSD17B10,BCAT2,ACADSB
Branched-chain α-keto acid Dehydrogenase Complex	5.E-02	5E-01	2/4 (50%)	BCKDHA,BCKDHB

Methylmalonyl Pathway	5.E-02	5E-01	2/4 (50%)	MCEE,PCCB
Glucocorticoid Biosynthesis	5.E-02	5E-01	2/4 (50%)	Cyp2d22,HSD3B7
Mineralocorticoid Biosynthesis	5.E-02	5E-01	2/4 (50%)	Cyp2d22,HSD3B7
Fatty Acid β-oxidation III (Unsaturated, Odd Number)	5.E-02	5E-01	2/4 (50%)	Eci3,ECI1
Interferon Signaling	5.E-02	2.27E-01	5/22 (23%)	IFIT3,IFITM3,PSMB8,TAP1,IRF1
Antigen Presentation Pathway	5.E-02	2.27E-01	5/22 (23%)	PSMB9,HLA-A,PSMB8,TAP1,TAP2

Table S4. The significant canonical pathways identified by downregulated differentially expressed genes (DEGs) in Taeeumjowuitang (TJ) versus high-fat-diet (HFD) for the epididymal white adipose tissue. Significant canonical pathways were obtained from Ingenuity Pathway Analysis. TJ= mice fed a high-fat-diet supplemented with Taeeumjowitang, HFD=mice fed a high-fat-diet.

Ingenuity Canonical Pathways	p-value	Ratio	Downregulated	Molecules
Leukocyte Extravasation Signaling	3.E-08	2.74E-01	32/117 (27%)	RAC2,MMP7,MMP3,ITGB3,EZR,CYBB,VCL,MMP12,MMP19,ATM,TIMP2,ITGA4,ARHGAP6,CXCR4,MMP2,NCF4,SELPLG,BTK,TEC,ITGB2,WIPF1,NCF1,ITGAM,CLDN8,WAS,PRKCD,PLCG2,NCF2,CD44,VAV1,PRKCH,PRKCB
Fcγ Receptor-mediated Phagocytosis in Macrophages and Monocytes	3.E-07	3.28E-01	21/64 (33%)	ACTR2,RAC2,FCGR2A,TLN1,FYB,INPP5D,MYO5A,HMOX1,PAK1,NCF1,ACTR3,WAS,EZR,ARPC2,PRKCD,SYK,HCK,AKT3,VAV1,PRKCH,PRKCB
Phagosome formation	2.E-06	3.4E-01	17/50 (34%)	MRC1,FN1,MSR1,FCGR2A,INPP5D,TLR2,TLR4,CLEC7A,PRKCD,PLCG2,SYK,TLR1,TLR6,PRKCH,ATM,ITGA4,PRKCB
Inhibition of Matrix Metalloproteases	4.E-05	4.5E-01	9/20 (45%)	MMP7,SDC1,ADAM12,MMP3,MMP2,MMP12,LRP1,TIMP2,MMP19
Axonal Guidance Signaling	9.E-05	1.82E-01	37/203 (18%)	RAC2,MMP7,TUBB,ADAM8,PAK1,ACTR3,MKNK1,ADAM19,AKT3,GNA13,GSK3B,LNPEP,RASA1,ITGA4,ATM,ACTR2,PLXNA1,STK36,PLXNC1,NRP2,CXCR4,GNG2,PRKAR2A,MMP2,SLIT2,WIPF1,SEMA4D,TUBB6,ADAM12,WAS,PRKCD,ARPC2,PLCG2,PRKCH,GNAL,FZD7,PRKCB
Role of Pattern Recognition Receptors in Recognition of Bacteria and Viruses	1.E-04	2.62E-01	17/65 (26%)	C3,C1QC,C1QA,C1QB,TLR2,TLR4,CLEC7A,TICAM1,PRKCD,PLCG2,SYK,TLR1,TLR6,CASP1,PRKCH,ATM,PRKCB
Granulocyte Adhesion and Diapedesis	1.E-04	2.35E-01	20/85 (24%)	MMP7,SDC1,MMP3,CXCR4,MMP2,ITGB3,GLG1,Ccl9,SE

				LPLG,ITGB2,ITGAM,CLDN8,IL1RN,EZR,Ccl2,CXCL14,MMP12,TNFRSF1B,MMP19,ITGA4
Actin Cytoskeleton Signaling	1.E-04	2.08E-01	25/120 (21%)	ACTR2,RAC2,TIAM1,FN1,MYH9,PIKFYVE,TLN1,IQGAP1,PAK1,ACTR3,CYFIP2,PPP1R12A,WAS,CYFIP1,FLNA,ARPC2,EZR,CD14,VAV1,GNA13,VCL,NCKAP1L,PIP4K2A,ATM,ITGA4
HER-2 Signaling in Breast Cancer	2.E-04	2.95E-01	13/44 (30%)	TSC1,ERBB3,MMP2,ITGB3,ITGB2,PRKCD,CDKN1A,AKT3,PRKCH,GSK3B,PRKCB,ATM,EGFR
TREM1 Signaling	2.E-04	3.08E-01	12/39 (31%)	TLR2,TLR4,Naip1 (includes others),TYROBP,PLCG2,NLRC3,TLR1,TLR6,CASP1,LAT2,AKT3,CD83
Complement System	2.E-04	4.21E-01	8/19 (42%)	ITGB2,ITGAM,C3,CFI,C1QC,C1QA,C1QB,CFH
Macropinocytosis Signaling	2.E-04	2.89E-01	13/45 (29%)	MRC1,ANKFY1,CSF1R,ITGB3,ITGB2,PAK1,HGF,PLCG2,PRKCD,CD14,PRKCH,ATM,PRKCB
Natural Killer Cell Signaling	4.E-04	2.59E-01	14/54 (26%)	RAC2,PTPN6,TYROBP,FCGR2A,INPP5D,PAK1,PRKCD,PLCG2,SYK,AKT3,PRKCH,VAV1,PRKCB,ATM
Rac Signaling	2.E-03	2.17E-01	15/69 (22%)	ACTR2,TIAM1,PIKFYVE,IQGAP1,PAK1,CYFIP2,ACTR3,CYFIP1,ARPC2,NCF2,CD44,CYBB,PIP4K2A,ATM,ITGA4
Agranulocyte Adhesion and Diapedesis	2.E-03	2E-01	18/90 (20%)	MMP7,MYH9,FN1,MMP3,CXCR4,MMP2,GLG1,Ccl9,SELPLG,ITGB2,CLDN8,IL1RN,EZR,Ccl2,CXCL14,MMP12,MMP19,ITGA4
Atherosclerosis Signaling	3.E-03	2.15E-01	14/65 (22%)	CCR3,MSR1,MMP3,CXCR4,CMA1,GLG1,SELPLG,COL1A2,ITGB2,LYZ,IL1RN,PLB1,ITGA4,COL3A1
G Beta Gamma Signaling	4.E-03	2.26E-01	12/53 (23%)	BTK,PAK1,PRKCD,PLCG2,GNG2,PRKAR2A,AKT3,PRKCH,GNA13,GNAL,EGFR,PRKCB
Protein Kinase A Signaling	4.E-03	1.56E-01	32/205 (16%)	UBASH3B,PTPRC,TGFBR2,PTPRJ,PPP1R10,PPP1R12A,FLNA,PTPRO,PTPN1,GNA13,GSK3B,PTPRE,PTPN6,ADCY3,GNG2,PRKAR2A,GYS2,PDE1C,AKAP13,PLCG2,PRKCD,CDC14B,EYA3,ITPR3,AKAP10,PTPRS,PRKCH,ADCY7,PTPRA,PTPN22,SIRPA,PRKCB
Hepatic Fibrosis / Hepatic Stellate Cell Activation	4.E-03	1.8E-01	20/111 (18%)	CCR5,COL5A2,MYH9,COL4A5,FN1,COL12A1,IL6R,MMP2,TGFBR2,COL1A2,TLR4,COL6A3,HGF,PDGFRA,CD14,TNFRSF1B,IFNAR1,EGFR,TIMP2,COL3A1
Virus Entry via Endocytic Pathways	5.E-03	2.18E-01	12/55 (22%)	ITGB2,RAC2,FLNA,PRKCD,PLCG2,CLTC,PRKCH,AP1B1,ITGA4,ATM,ITGB3,PRKCB

Colorectal Cancer Metastasis Signaling	5.E-03	1.68E-01	23/137 (17%)	MMP7,JAK1,MMP3,ADCY3,GNG2,IL6R,PRKAR2A,MMP2,TGFBR2,TLR2,TLR4,CDH1,TLR1,TLR6,AKT3,GSK3B,MMP12,ADCY7,LRP1,MMP19,EGFR,ATM,FZD7
D-myo-inositol-5-phosphate Metabolism	7.E-03	1.92E-01	15/78 (19%)	PTPN6,MTMR9,PTPRC,TNS3,ATP1A1,PTPRJ,PPP1R12A,PTPRO,PLCG2,PTPN1,EYA4,PIP4K2A,RASA1,PTPN22,SIRPA
3-phosphoinositide Biosynthesis	7.E-03	1.86E-01	16/86 (19%)	PTPN6,MTMR9,PIKFYVE,PTPRC,TNS3,ATP1A1,PPP1R12A,PTPRJ,PTPRO,PTPN1,EYA4,PIP4K2A,RASA1,PTPN22,SIRPA,ATM
DNA Double-Strand Break Repair by Non-Homologous End Joining	7.E-03	4.44E-01	4/9 (44%)	DCLRE1C,PARP1,ATM,LIG3
BER pathway	7.E-03	4.44E-01	4/9 (44%)	LIG1,PCNA,PARP1,LIG3
Cell Cycle: G2/M DNA Damage Checkpoint Regulation	7.E-03	2.58E-01	8/31 (26%)	CDKN1A,TRIP12,TOP2A,RPS6KA1,HIPK2,BRCA1,SKP2,ATM
Tec Kinase Signaling	8.E-03	1.81E-01	17/94 (18%)	JAK1,GNG2,TEC,BTK,TLR4,PAK1,WAS,PRKCD,PLCG2,HCK,PRKCH,VAV1,GNA13,GNAL,ATM,ITGA4,PRKCB
Agrin Interactions at Neuromuscular Junction	8.E-03	2.27E-01	10/44 (23%)	LAMC1,ITGB2,RAC2,PAK1,PKLR,ERBB3,DAG1,ITGA4,EGFR,ITGB3
Superpathway of Inositol Phosphate Compounds	8.E-03	1.76E-01	18/102 (18%)	PTPN6,MTMR9,PIKFYVE,INPP5D,PTPRC,TNS3,ATP1A1,PPP1R12A,PTPRJ,PTPRO,PLCG2,PTPN1,EYA4,PIP4K2A,RASA1,PTPN22,SIRPA,ATM
Production of Nitric Oxide and Reactive Oxygen Species in Macrophages	8.E-03	1.73E-01	19/110 (17%)	PTPN6,JAK1,NCF4,TLR2,TLR4,NCF1,LYZ,PPP1R12A,PPP1R10,PRKCD,PLCG2,NCF2,CYBB,AKT3,PRKCH,TNFRSF1B,SIRPA,ATM,PRKCB
Signaling by Rho Family GTPases	9.E-03	1.63E-01	22/135 (16%)	ACTR2,GNG2,PIKFYVE,IQGAP1,PKN1,CDH11,PAK1,CDH1,WIPF1,ACTR3,PPP1R12A,WAS,CYFIP1,EZR,ARPC2,NCF2,CYBB,GNA13,PIP4K2A,GNAL,ATM,ITGA4
PI3K Signaling in B Lymphocytes	1.E-02	1.8E-01	16/89 (18%)	BLNK,ATF3,C3,ATF7,INPP5D,BTK,PTPRC,TLR4,CD180,PLCG2,SYK,ITPR3,AKT3,VAV1,PIK3AP1,PRKCB
Regulation of Actin-based Motility by Rho	1.E-02	2.08E-01	11/53 (21%)	RAC2,ACTR2,WIPF1,PAK1,ACTR3,PPP1R12A,WAS,ARPC2,PIKFYVE,PIP4K2A,ITGA4
MSP-RON Signaling Pathway	1.E-02	2.59E-01	7/27 (26%)	TLR2,CSF2RB,ITGB2,TLR4,ITGAM,RPS6KA2,ATM
Remodeling of Epithelial Adherens Junctions	1.E-02	2.25E-01	9/40 (23%)	ACTR2,CDH1,ACTR3,TUBB6,ARPC2,HGF,VCL,TUBB,IQGAP1
Epithelial Adherens Junction Signaling	1.E-02	1.79E-01	15/84 (18%)	ACTR2,MYH9,BMPR2,IQGAP1,TUBB,TGFBR2,CDH1,AC

				TR3,TUBB6,WAS,ARPC2,HGF,AKT3,VCL,EGFR
IL-8 Signaling	1.E-02	1.64E-01	19/116 (16%)	RAC2,GNG2,MMP2,IQGAP1,ITGB3,ITGB2,HMOX1,CDH1,ITGAM,PRKCD,NCF2,CYBB,ITGAV,AKT3,PRKCH,GNA13,EGFR,ATM,PRKCB
IL-3 Signaling	1.E-02	2.08E-01	10/48 (21%)	CSF2RB,PTPN6,PAK1,JAK1,PRKCD,AKT3,PRKCH,INPP5D,ATM,PRKCB
CXCR4 Signaling	1.E-02	1.76E-01	15/85 (18%)	CXCR4,ADCY3,GNG2,PAK1,PRKCD,ITPR3,AKT3,PRKCH,GNA13,ELMO2,ADCY7,ELMO1,GNAL,PRKCB,ATM
phagosome maturation	1.E-02	1.86E-01	13/70 (19%)	VPS18,TUBB6,ATP6V1C1,TCIRG1,NCF2,ATP6V0A2,PIKFYVE,CYBB,ATP6V1A,TUBB,ATP6V0A1,ATP6V1B2,EEA1
DNA Double-Strand Break Repair by Homologous Recombination	2.E-02	3.64E-01	4/11 (36%)	LIG1,POLA1,BRCA1,ATM
γ-linolenate Biosynthesis II (Animals)	2.E-02	3.64E-01	4/11 (36%)	ACSBG1,SLC27A2,FADS2,ACSL4
Mitochondrial L-carnitine Shuttle Pathway	2.E-02	3.64E-01	4/11 (36%)	ACSBG1,CPT1A,SLC27A2,ACSL4
fMLP Signaling in Neutrophils	2.E-02	1.83E-01	13/71 (18%)	ACTR2,GNG2,NCF1,ACTR3,WAS,ARPC2,PRKCD,ITPR3,NCF2,CYBB,PRKCH,ATM,PRKCB
Integrin Signaling	2.E-02	1.59E-01	20/126 (16%)	ACTR2,RAC2,PIKFYVE,TLN1,ITGB3,ITGB2,PAK1,WIPF1,ACTR3,ITGAM,PPP1R12A,WAS,PLCG2,ARPC2,ITGAV,AKT3,VCL,GSK3B,ATM,ITGA4
Molecular Mechanisms of Cancer	2.E-02	1.44E-01	29/201 (14%)	RAC2,Naip1 (includes others),JAK1,BMPR2,TGFBR2,PAK1,FANCD2,AKT3,GSK3B,GNA13,BRCA1,HIPK2,RASA1,ATM,ITGA4,NAIP,STK36,ADCY3,APAF1,PRKAR2A,CDH1,PRKCD,CDKN1A,PRKCH,ADCY7,GNAL,LRP1,PRKCB,FZD7
RhoGDI Signaling	2.E-02	1.67E-01	17/102 (17%)	ACTR2,ARHGAP6,GNG2,PIKFYVE,CDH11,DGKZ,PAK1,CDH1,ACTR3,PPP1R12A,ARPC2,EZR,CD44,GNA13,PIP4K2A,GNAL,ITGA4
NF-κB Signaling	2.E-02	1.64E-01	18/110 (16%)	TRAF3,BMPR2,IGF2R,TGFBR2,TLR2,TLR4,TNIP1,IL1RN,PLCG2,TLR6,TLR1,PDGFRA,AKT3,GSK3B,TNFRSF1B,EGFR,ATM,PRKCB
α-Adrenergic Signaling	2.E-02	1.93E-01	11/57 (19%)	PRKCD,PLCG2,GNG2,ITPR3,ADCY3,PRKAR2A,PRKCH,SLC8A1,ADCY7,PRKCB,GYS2
D-myo-inositol (1,4,5,6)-Tetrakisphosphate Biosynthesis	2.E-02	1.81E-01	13/72 (18%)	PTPN6,MTMR9,PTPRC,ATP1A1,TNS3,PPP1R12A,PTPRJ,PTPRO,PTPN1,EYA4,RASA1,PTPN22,SIRPA
D-myo-inositol (3,4,5,6)-tetrakisphosphate	2.E-02	1.81E-01	13/72 (18%)	PTPN6,MTMR9,PTPRC,ATP1A1,TNS3,PPP1R12A,PTPRJ,P

Biosynthesis				TPRO,PTPN1,EYA4,RASA1,PTPN22,SIRPA
Caveolar-mediated Endocytosis Signaling	2.E-02	2E-01	10/50 (20%)	ITGB2,ITGAM,FLNA,PTPN1,ITGAV,CD48,DYRK3,ITGA4,EGFR,ITGB3
NF-κB Activation by Viruses	2.E-02	2E-01	10/50 (20%)	ITGB2,CCR5,PRKCD,ITGAV,AKT3,PRKCH,ITGA4,ATM,ITGB3,PRKCB
Cdc42 Signaling	2.E-02	1.85E-01	12/65 (18%)	ACTR2,WIPF1,PAK1,ACTR3,PPP1R12A,WAS,ARPC2,VAV1,GSK3B,IQGAP1,RASA1,ITGA4
GPCR-Mediated Nutrient Sensing in Enteroendocrine Cells	2.E-02	2.05E-01	9/44 (20%)	PRKCD,PLCG2,GNG2,ITPR3,ADCY3,PRKAR2A,PRKCH,ADCY7,PRKCB
3-phosphoinositide Degradation	2.E-02	1.71E-01	14/82 (17%)	PTPN6,MTMR9,INPP5D,PTPRC,ATP1A1,TNS3,PPP1R12A,PTPRJ,PTPRO,PTPN1,EYA4,RASA1,PTPN22,SIRPA
Fc Epsilon RI Signaling	3.E-02	1.83E-01	11/60 (18%)	BTK,RAC2,PRKCD,PLCG2,SYK,AKT3,VAV1,PRKCH,INPP5D,ATM,PRKCB
Stearate Biosynthesis I (Animals)	3.E-02	2.4E-01	6/25 (24%)	ACSBG1,SLC27A2,FASN,ELOVL2,ACSL4,ELOVL6
FcγRIIB Signaling in B Lymphocytes	3.E-02	2.31E-01	6/26 (23%)	BLNK,BTK,PLCG2,SYK,INPP5D,ATM
Ephrin B Signaling	3.E-02	2E-01	8/40 (20%)	RAC2,PAK1,CXCR4,GNG2,ITSN2,VAV1,GNA13,GNAL
Fatty Acid Activation	3.E-02	3.75E-01	3/8 (38%)	ACSBG1,SLC27A2,ACSL4
Communication between Innate and Adaptive Immune Cells	3.E-02	2.12E-01	7/33 (21%)	TLR2,TLR4,IL1RN,TLR1,TLR6,CD83,Ccl9
GADD45 Signaling	4.E-02	2.86E-01	4/14 (29%)	PCNA,CDKN1A,BRCA1,ATM
RhoA Signaling	4.E-02	1.67E-01	12/72 (17%)	PLXNA1,ACTR2,ARHGAP6,ACTR3,NRP2,PPP1R12A,ARPC2,EZR,PIKFYVE,GNA13,PIP4K2A,PKN1
B Cell Receptor Signaling	4.E-02	1.52E-01	16/105 (15%)	BLNK,RAC2,PTPN6,FCGR2A,INPP5D,BTK,PTPRC,PLCG2,SYK,CD22,AKT3,VAV1,PIK3AP1,GSK3B,ATM,PRKCB
Endothelin-1 Signaling	4.E-02	1.57E-01	14/89 (16%)	ADCY3,MAPK6,HMOX1,PLB1,PRKCD,PLCG2,ITPR3,CASP1,PRKCH,GNA13,ADCY7,GNAL,PRKCB,ATM
Gαq Signaling	4.E-02	1.57E-01	14/89 (16%)	RGS2,RGS18,GNG2,GYS2,BTK,HMOX1,PRKCD,PLCG2,ITPR3,AKT3,PRKCH,GSK3B,PRKCB,ATM
Growth Hormone Signaling	4.E-02	1.9E-01	8/42 (19%)	PTPN6,PRKCD,PLCG2,PRKCH,RPS6KA2,RPS6KA1,ATM,PRKCB
14-3-3-mediated Signaling	4.E-02	1.69E-01	11/65 (17%)	TSC1,TUBB6,PRKCD,PLCG2,AKT3,PRKCH,RPS6KA1,GSK3B,TUBB,ATM,PRKCB
Melanocyte Development and Pigmentation Signaling	5.E-02	1.8E-01	9/50 (18%)	PTPN6,MITF,PLCG2,ADCY3,PRKAR2A,RPS6KA2,RPS6KA1,ADCY7,ATM

ErbB Signaling	5.E-02	1.8E-01	9/50 (18%)	PAK1,PRKCD,PLCG2,ERBB3,PRKCH,GSK3B,ATM,EGFR,PRKCB
Cholesterol Biosynthesis I	5.E-02	3.33E-01	3/9 (33%)	SQLE,NSDHL,LSS
Cholesterol Biosynthesis II (via 24,25-dihydrolanosterol)	5.E-02	3.33E-01	3/9 (33%)	SQLE,NSDHL,LSS
Cholesterol Biosynthesis III (via Desmosterol)	5.E-02	3.33E-01	3/9 (33%)	SQLE,NSDHL,LSS
DNA damage-induced 14-3-3σ Signaling	5.E-02	3.33E-01	3/9 (33%)	AKT3,BRCA1,ATM
VDR/RXR Activation	5.E-02	1.86E-01	8/43 (19%)	SPP1,PRKCD,CDKN1A,NCOA1,CD14,PRKCH,NCOA3,PRKCB
